# Anorexia nervosa – a mask or a prelude to schizophrenia and bipolar disorder in adolescents

**DOI:** 10.1192/j.eurpsy.2026.11113

**Published:** 2026-07-31

**Authors:** W. Wójtowicz, Z. Mielczarek, M. Wadoń, M. Bień, M. Janas-Kozik

**Affiliations:** 1 Student Scientific Society at the Department of Psychiatry and Psychotherapy of Developmental Age; 2 Department and Clinical Ward of Psychiatry and Psychotherapy of Developmental Age, Medical University of Silesia, Katowice, Poland

## Abstract

**Introduction:**

Anorexia nervosa affects 0.3–1% of the general population and occurs more often in patients with schizophrenia (1–4%), frequently emerging in the prodromal phase. Body image distortion and food restriction may precede psychotic symptoms and sometimes subside as schizophrenia progresses. Anorexia is viewed by some as an early cognitive marker or as coexisting with psychosis, with delusions, obsessive thoughts, and negative symptoms being common.

Mood disturbances are also frequent in eating disorders. In bipolar disorder, impaired impulse control may link the two conditions. While bipolar disorder affects about 1% of the population, anorexia nervosa is reported in 3–25% of such patients.

**Objectives:**

This study aims to analyze a series of clinical cases involving female patients in whom anorexia nervosa symptoms either preceded or co-occurred with schizophrenia and bipolar disorder, highlighting potential links between these disorders in terms of comorbidity and underlying pathophysiological mechanisms.

**Methods:**

This study is based on a series of three case reports. Clinical data were collected retrospectively through a detailed review of available hospital records, including admission notes, psychiatric evaluations, and follow-up documentation. The analysis focused on the course of illness, diagnostic revisions, and the relationship between anorexia nervosa, schizophrenia, and bipolar disorder in adolescent patients.

**Results:**

Three adolescent females initially diagnosed with anorexia nervosa later developed mood and psychotic symptoms, prompting revised diagnoses. A 15-year-old with BMI 13.4 and mood elevation was reclassified as bipolar disorder. A 14-year-old with BMI 14.7, self-injury, and psychotic depression progressed to bipolar disorder and ultimately paranoid schizophrenia. A 13-year-old with BMI 16.8 and depressive features developed suicidal ideation and somatic delusions, leading to paranoid schizophrenia. These cases highlight diagnostic overlap between anorexia nervosa, mood disorders, and schizophrenia in adolescence, underscoring the need for longitudinal reassessment.

**Conclusions:**

Anorexia nervosa may emerge during the prodromal phase or coexist with schizophrenia and bipolar disorder. It can serve as a clinical mask of these disorders, manifesting as a delusional equivalent in schizophrenia or as a marker of impaired impulse control in bipolar disorder. Emotional and mood-related symptoms observed in anorexia nervosa may reflect negative symptoms of schizophrenia or represent features of depressive or hypomanic episodes in bipolar disorder. Diagnostic challenges arise from the fact that anorexia nervosa may represent a prodromal stage, a symptomatic equivalent, or an independent but co-occurring condition in relation to schizophrenia and bipolar disorder.

**Disclosure of Interest:**

None Declared

